# The Formation and Therapeutic Update of Tumor-Associated Macrophages in Cervical Cancer

**DOI:** 10.3390/ijms20133310

**Published:** 2019-07-05

**Authors:** Qun Wang, Alexander Steger, Sven Mahner, Udo Jeschke, Helene Heidegger

**Affiliations:** 1 Department of Obstetrics and Gynecology, University Hospital, LMU Munich, 80337 Munich, Germany; 2 Klinik für Innere Medizin I, Technische Universität München, 80333 Munich, Germany

**Keywords:** tumor-associated macrophages, cervical cancer, biomarker, T cell, seminal plasma, anaerobic microenvironment

## Abstract

Both clinicopathological and experimental studies have suggested that tumor-associated macrophages (TAMs) play a key role in cervical cancer progression and are associated with poor prognosis in the respects of tumor cell proliferation, invasion, angiogenesis, and immunosuppression. Therefore, having a clear understanding of TAMs is essential in treating this disease. In this review, we will discuss the origins and categories of macrophages, the molecules responsible for forming and reeducating TAMs in cervical cancer (CC), the biomarkers of macrophages and the therapy development targeting TAMs in CC research.

## 1. Introduction

Although vaccinations, radical operations, and radiochemotherapy have been widely used for preventing and treating cervical cancer (CC), there are still over half a million new cases worldwide annually. CC is the fourth most common cause of death from cancer among women [1]. Persistent infection of human papillomavirus (mainly HPV16 and HPV18) in the cervix has been established as the primary cause of cervical cancer [2]. E6 and E7, as HPV oncogenes, contribute to neoplastic progression when combined with the host cervical cell genome [3]. Drug resistance, recurrence, and metastasis are the primary culprits for increased mortality, which are also associated with the tumor microenvironment (TME). The TME is mainly compromised of tumor cells, bone-marrow-derived cells and host stromal cells. Their components interact to offer an immunosuppressive environment, thus facilitating tumor progression [4]. Among these components, the role of tumor-associated macrophages (TAMs) has been receiving increasing consideration. TAMs belong to a subunit of macrophages that are recruited to tumor tissue and affect tumor growth [5]. Dr. Günther pioneered the macrophage electrophoresis mobility test (MEM test) in the diagnosis of malignant gynecological diseases [6]. As a result, the function of TAMs in CC has attracted more and more attention in recent years [7]. In this review, we will discuss the origins and categories of macrophages, the molecules involved in forming and reeducating TAMs in CC, the biomarkers of macrophages and the therapy development targeting TAMs in CC research.

## 2. The Origins and Categories of Macrophages

There are two kinds of macrophages involved in the pathophysiology of cervical cancer: tissue-resident macrophages and infiltrating macrophages (Table 1).

### 2.1. Tissue-Resident Macrophages

During embryonic organogenesis, macrophages derived from yolk sac and fetal liver precursors are seeded in or on the epithelia throughout normal, non-injured or inflamed tissues. These macrophages persist into adulthood as resident, self-maintaining populations [8]. They generally show specialized properties and functions that are related to their respective tissue locations, such as Kupffer cells in the liver, alveolar macrophages in the lungs, red pulp macrophages in the spleen and microglia in the brain [12,13]. After birth, bone marrow or spleen-derived monocytes can replenish tissue resident macrophages following injury, infection or inflammation. These macrophages reside in interstitial locations within the tissue. For example, the replacement of alveolar macrophages by postnatal monocytes yielded new macrophages that had an expression profile that was almost identical to the original embryonically derived alveolar macrophages. This suggests an overarching role of the tissue environment in dictating overall macrophage function [9]. Tissue-resident macrophage maintenance and homeostasis depend on its origin in the steady state, which can be disturbed by pathological or physiological inflammation [8,14]. For example, small doses of human seminal plasma inhibited lymphocyte transformation, which could possibly be mediated through accessory macrophages. Investigators also revealed that seminal plasma interferes with the attachment, spreading and phagocytic activity of C. parvum elicited murine peritoneal macrophages [15]. Moreover, treatment with Sizofiran (SPG) and recombinant interferon γ (IFN-γ) could make peritoneal macrophages obtained from CC acquire new potent tumoricidal activity along with the increasing secretion of TNF, IFN-γ, and IL-1 and inhibiting the increased secretion of PGE2 [16].

### 2.2. Infiltrating Macrophages

Many pro-inflammatory mediators recruit circulating monocytes to tumor, inflammatory or infected tissue and acquire the properties of infiltrating macrophages. Along with the inflammatory stage, inflammatory macrophages present mainly two types of properties, ‘pro-inflammatory’ versus ‘pro-resolving’, ‘classically activated’ versus ‘alternatively activated’ or ‘M1-like’ versus ‘M2-like’. These properties are under the influence of growth factors, metabolic requirements, local oxygen tension, the tissue cells and the tissue matrix [10]. In fact, monocyte-derived macrophages in the tissue are often highly heterogeneous as they go through the various influencing factors. For example, four subsets of M2 were identified, including M2a, M2b, M2c, and M2d [17].

M1-like polarized macrophages are induced by Th1 cytokines IFN γ, IL-2, IL-3, IL-12, TNF-α, bacterial constituent such as lipopolysaccharide (LPS), and Toll-like receptor (TLR) agonists. M1-like macrophages secret pro-inflammatory factors such as IL-1 beta, IL-6, IL-12, IL-23, iNOS and tumor necrosis factor-α (TNF-α), chemokine ligands 9 (CXCL-9), CXCL-10 and express the major histocompatibility complex class I (MHC I) and class II (MHC II) molecules. Thus, the M1-like macrophages act in the inflammatory response and antitumor immunity. Oppositely, the M2-like macrophages are induced by Th2 cytokines such as IL-4, IL-10, IL-13, TNF-α, TGF-β, GM-CSF, immune complexes and TLRs. M2-like macrophages secret a wide array of anti-inflammatory molecules, such as IL-10, TGF-β, and arginase 1 and exert anti-inflammatory and pro-tumorigenic activities [11,18]. TAMs could promote the proliferation, invasion, and metastasis of cervical cancer cells. In addition, TAMs could participate in tissue remodeling, fibrosis and angiogenesis in cervical cancer. Recent studies show TAMs exhibit functions similar to those of M2-like macrophages [19].

## 3. The Molecules Involved in Forming TAMs

In cervical cancer, the mechanism involved in forming TAMs mainly relate to tumor-derived molecules, T cell-derived molecules, seminal plasma-derived molecules, the anaerobic microenvironment and molecules from other sources.

### 3.1. Tumor-Derived Molecules

Multiple malignant tumor cell-derived molecules could promote TAM formation. For example, the conditioned medium of pancreatic cells, which contained secreted REG4, could induce the polarization of macrophages to an M2-like phenotype [20]. Prostate cancer cells secreted CCN3, which could recruit macrophages and skew their differentiation to an M2-like phenotype [21]. Meanwhile, the supernatant of colorectal cancer cells induced the M2-like polarization of macrophages [22]. Finally, both senescent thyrocytes and thyroid tumor cell lines triggered M2-like macrophage polarization that was related to PGE2 secretion [23].

As to the formation of TAMs in cervical cancer, in 1982 investigators noted that monocyte functions of chemotaxis, phagocytosis and helper function on T-cell mitogen response were significantly inhibited when monocytes were pre-incubated in cervical cancer sera. It was speculated that some components in cancer sera played an immuno-suppressive role through the inhibition of monocyte functions in cancer-bearing state [24]. It was further discovered that when treated with the supernatant of CC cell lines, M1-like macrophages developed an M2-like phenotype with increased CD163, TLR-3, -7, -9 and IL-10 [25,26]. Similarly, co-culture cervical cancer cells decrease the macrophage M1-like polarization partly through necroptosis downregulation [27]. However, these studies did not clarify which component of the supernatant was responsible for the M2-like induction. With the development of research, investigators have found that CC cell-derived molecules act in the polarization and activation of monocytes (Table 2).

#### 3.1.1. Molecules Promoting the Differentiation of Monocytes to TAMs

PGE2 and IL-6 produced by CC cells could cause M2-like phenotype differentiation. Furthermore, these M2-like macrophages displayed a lower expression of costimulatory molecules, an altered balance in IL-12p70 and IL-10 production, and a poor capacity to stimulate T-cell proliferation and IFN-γ production after having been activated by TLR-agonists. This creates a tolerogenic tumor microenvironment [28].

Karyopherin β1 is a nuclear import protein involved in the transport of proteins containing a nuclear localization sequence. Transcriptional factors such as NFκB and AP-1 initiate the expression of multiple factors associated with inflammation and cancer cell biology. Inhibition of Karyopherin β1 in HeLa cells led to declined transcriptional activity of NFκB and AP-1 and reduced migration and invasion of cervical cancer cells [29]. In addition, a study showed that E6 stimulated the expression of multiple genes known to be inducible by NFkB and AP-1 [38], such as COX-2 [39]. The onocogene E6 enhanced expression of functional components of the NFκB signal pathway, including p50, NIK, and TRAF-interacting protein, and increased the binding of NFκB and AP-1 to DNA consensus binding sites [38]. Also, Th2 cell- secreting cytokines such as interleukin-6, interleukin-1β, TNFα, and GM-CSF are also the targeted genes of NFκB and AP-1 [29]. It was further found that Th2 cytokines induce M2-like macrophages. [18]. These results indicated that Karyopherin β1 may participate in inducing M2-like macrophage differentiation, but the existing studies have lacked direct evidence to show the effect of Karyopherin β1 on macrophage polarization.

The chemokine CCL2 (MCP-1) combines with the receptors on monocytes and is important for monocyte recruitment and modulation of macrophages towards the M2-like phenotype. CCL2 was found to determine the extent of macrophage polarization because CCL2 enhanced the LPS-induced production of IL-10, whereas the blockade of CCL2 led to enhanced expression of M1-like polarization-associated genes and cytokines, and diminished expression of M2-like-associated markers in human macrophages [28,29,30,38]. The CCL2 mRNA expression level in CC cells was positively associated with the number of TAMs. Lack of CCL2 mRNA was associated with increased cumulative relapse-free survival, cumulative overall survival, less post-operative surgery, reduced local and distant recurrence, reduced vascular invasion and smaller tumor size [40].

IL-10 is produced by multiple cells, including HPV transformed CC cells [41]. IL-10 causes contradictory effects on CC. It has been found that high IL-10 levels may prevent cervical neoplasia by assisting HPV elimination [42]. Conversely, low IL-10 levels are associated with a higher risk for cervical cancer [43]. It could be that IL-10 induces monocytes to the M2c subtype [44], while also inhibiting MHC I and MHC II expression and thus impairing their ability to present antigens. [45,46,47]. Other research has shown that IL-10 inhibited the classic activation of macrophages by JAK1/Tyk2/STAT3 pathway, which was the most commonly activated pathway by IL-10 [31].

A variant of prolactin (PRL) is produced by cervical cancer cells. It reduced apoptosis in HeLa, SiHa and C-33A cell lines and induced IL-1β and TNF-α production by human peripheral blood mononuclear cell line THP-1 macrophages [32]. Higher doses of PRL (1000 ng/mL) induces macrophages to produce anti-inflammatory cytokine IL-10, which leads to a significant decrease in production of proinflammatory cytokines [33]. These results indicated that PRL might participate in the polarization of monocytes towards the M2-2 phenotype.

#### 3.1.2. Molecules Promoting the Activation of TAMs

α-l-Fucosidase (FUCA-1) activity was found to be increased in CC tissue. FUCA-1 is a glycosidase, which splits terminal α-l-fucose from glycoproteins [48]. FUCA-1 was discovered to be expressed in thyroid carcinoma cell lines. However, few research papers have reported its expression in CC cells. After incubation of human monocyte-derived macrophages with FUCA-1, the macrophages were no longer able to respond to lipopolysaccharide (LPS). In addition, after tumor cells were treated with FUCA-1 and then co-cultivated with macrophages, macrophage-mediated cytotoxicity had no effect on tumor cells [34]. These studies indicate that FUCA-1 may be over-expressed in CC cells and might promote the activation of TAMs by inhibiting antigen presentation function and phagocytosis.

Cervical cancer progression is associated with increased serum levels of granulocyte macrophage colony-stimulating factor (GM-CSF) [49]. The expression levels of GM-CSF produced by CC cells were positively correlated with the number of TAMs [50]. CC cells release GM-CSF, which in turn activates mononuclear phagocytes and induces them to release HB-EGF. HB-EGF then triggers anti-apoptotic and proliferative signals in CC cells, which leads to the release of more GM-CSF [35]. These studies indicate that GM-CSF could activate TAMs to release pro-tumor factors.

Macrophage migration inhibitory factor (MIF) expression was significantly increased in cervical cancer samples. SiHa and CaSki cervical cancer cells were discovered to secret soluble MIF into cell culture supernatants [51]. CD74, a receptor for MIF, has been known to be expressed on HLA Class II-positive normal cells including macrophages [52]. The genetic polymorphism MIF-173 is associated with cervical cancer in humans. Patients with the CC genotype exhibited higher MIF serum concentration, which could increase the risk of early stage cervical cancer and lymphatic metastasis [53]. Deep stromal infiltration correlated with the overexpression of MIF in cervical intraepithelial neoplasia (CIN) and squamous cell carcinoma (SCC). Both protein levels of MIF and CD74 were also associated with microvessel density [54]. What is more, long-term loss of MIF significantly inhibited growth and proliferation of HeLa cells while increasing HeLa cell adhesion and therefore impairing their migratory capacity [55]. In melanomas, MIF deficiency attenuated the alternative activation of tumor-polarized macrophages, immunosuppression, and neoangiogenesis [36]. These studies indicate that MIF may participate in the activation of M2-like macrophages in CC.

IL-10 is produced by multiple cells, including HPV transformed CC cells [41] and TAMs [37]. In tuberculosis, macrophage-derived IL-10 could promote M2-like activation by elevating the expression of IL-4Rα as well as IL-4R-dependent arginase 1 [37]. Moreover, IL-10 could promote the expression of arginase with LPS so that the macrophages could exert the ability of immunosuppression [56,57]. These studies implicate that IL-10 from CC cells may participate in the alternative activation of macrophages.

#### 3.1.3. The role of Oncoproteins on TAMs Formation in CC

**Figure 1:**
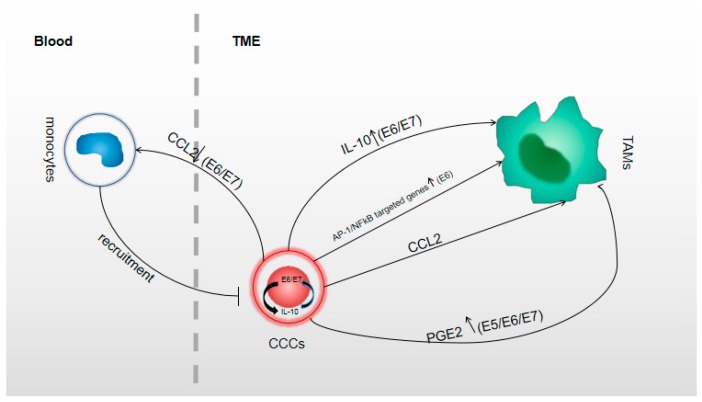
This figure shows the relationship between human papillomavirus (HPV) oncoproteins and the molecules from CC cells acting in formatting TAMs. In circulating blood, CCL2 (left) was regulated inversely by E6 or E7 [64], and the less expressed CCL2 recruited fewer monocytes into local tumor tissue [30]; IL-10 was upregulated by E6 or E7 and E6 and E7 were upregulated by IL-10 as a circulation in CC cells [65]; Increasing IL-10 could act in M2 polarization process [44]; Transcriptional factors AP-1 and NFκB targeted genes that act in M2 polarization were upregulated by E6 [38]; CCL2 (right) was expressed individualized [30,40,44,66] and CCL2^+^ CC cells could promote the M2 polarization [30,40]. The molecules which regulated CCL2 expression that act in M2 polarization was unclear. PGE2 which acted in M2 polarization was upregulated by E5 or E6 or E7 [67,68]; TME, tumor microenvironment; CCL2 is the alternative name of monocyte chemoattractant protein (MCP-1); IL-10, interleukin 10; CC cells, cervical cancer cells.

The HPV genome contains eight open reading frames (ORF) divided into two regions, the early (E) and the late (L) regions. The E-region encodes for six genes (E1, E2, E4, E5, E6, and E7) [58]. Generally, E1, E2, and E4 proteins are responsible for viral amplification and release [59,60,61]. E5, E6 and E7 proteins show tumor-promoting activities, with E6 and E7 corresponding to the primary transformation of viral proteins [61,62,63]. Oncoproteins could promote the production of molecules which participate in TAM formation in CC (Figure 1).

Accumulated data has shown that after HPV infection, IL-10 levels increase because HPV E2, E6, and E7 proteins act in IL10 gene transcription, while IL-10 stimulates HPV E6 and E7 expression [65]. Moreover, IL-10 participates in the differentiation of cells into M2-like macrophages. M2 macrophages were further characterized by expressing high levels of IL-10 as stated above [37,45,46,47,69]. Therefore, the interaction between HPV oncoproteins and IL-10 creates a vicious cycle that could favor an immunosuppressive microenvironment in the cervix.

The HPV-16 E6 protein stimulated the expression of multiple genes known to be inducible by NF-κB and AP-1. What is more, E6 enhanced expression of functional components within the NF-κB signal pathway [38]. The E6-dependent c-fos oncogenic protein expression contributes as well to AP-1 complex formation under oxidative stress in SiHa cells [70]. This leads to the idea that downstream targeted genes of NF-κB and AP-1 might participate in the differentiation towards the M2-like phenotype.

While several studies have shown that CCL2 participates in the recruitment of monocytes and promotes the polarization of monocytes into the M2-like phenotype, it should also be noted that the expression of CCL2 in CC cells was individualized [28,29,38,39]. Meanwhile, other studies have proven that the HPV oncogenes E6 or E7 inversely correlate with the expression of the *MCP-1* gene [69], which means E6 or E7 could inhibit the CCL2s ability to recruitment monocytes. There may, however, be other molecules in CC cells that positively regulate CCL2 expression.

E5, E6 or E7 could induce cyclooxygenase-2 (COX-2) expression which leads to increased PGE2 secretion. Among them, HPV-16 E6 and E7 oncoproteins induce COX-2 transcription by inducing the release of EGFR ligand amphiregulin and then activating the epidermal growth factor receptor (EGFR)-Ras-mitogen protein kinase pathway [67,68]. As earlier asserted, PGE2 has a predominant impact on the phenotype of the M2-like macrophage [28].

### 3.2. T Cell-Derived Molecules

T cells, such as CD4+ and T-regs, infiltrate the tissue in cervical cancer [71]. By secreting specific molecules, it could be that T cells affect the differentiation of monocytes to M2-like macrophages (Table 3).

IL-17 is a cytokine produced by an activated human memory CD4 T-cell Two cervical cell lines transfected with a cDNA encoding IL-17 exhibited a significant increase in tumor size. IL-17 increased IL-6 and IL-8 secretion in cervical carcinoma cell lines. This enhanced tumor growth elicited by IL-17 was associated with increased expression of IL-6 and also macrophage recruitment at the tumor site [72]. Meanwhile, IL-6 has a profound impact on the phenotype of M2-like macrophages [28]. The results implicate that IL-17 may be involved in the differentiation of infiltrating macrophages.

In CC, tumor infiltrating T-effector-cells constitutively expressed IL-4 [79]. IL-4 has been verified to promote bone marrow-derived macrophages (BMDMs) to polarize into the M2a-like subset by inducing a transcriptional factor Kruppel-like factor 4 (KLF4) which then acts in macrophage polarization [73]. Therefore, T-cell derived IL-4 may exert the same role in CC. In addition, IL-10 could induce monocytes to the M2c-like subtype [44]. IL-10 is produced by multiple cells, including T-regs [80]. Low IL-10 levels are associated with increased risk for cervical cancer [43]. So, T-cell derived IL-10 may activate the alternative activation of macrophages in CC. Recently a study has proven that the combination of IL-4 and IL-10 simulate macrophages to the M2a macrophage subtype [74].

### 3.3. Seminal Plasma-Derived Molecules

Seminal plasma (SP) could activate cyclooxygenase-2 and prostaglandin E2 receptor expression and promote the expression of tumorigenic and angiogenic genes via the E-series prostanoid 4 receptor in cervical adenocarcinoma cells [75,76]. In vitro stimulation of cervical cells with normal seminal plasma resulted in significantly elevated concentrations of secreted IL-6, IL-8 and GM-CSF [77]. Seminal plasma interfered with the attachment, spreading and phagocytic activity of C. parvum elicited murine peritoneal macrophages and the release of reactive oxygen species from zymosan triggered human peripheral blood monocytes [15]. Moreover, prostaglandin E2 and IL-6 acted in M2-like macrophage polarization [28]. These results indicate that SP may affect the differentiation of monocytes via prostaglandin in CC (Table 3).

### 3.4. Anaerobic Microenvironment

The hypoxic cervical TME stimulates the recruited macrophages to transform into the M2-like phenotype. Overexpressed Nrp-1 in hypoxia-primed cervical cancer cells was necessary for hypoxic cervical TME to recruit and polarize macrophages towards the M2-like phenotype. Nrp-1 and M2-like TAMs have been shown to be related to the malignant properties of cervical cancer, such as the FIGO stage and lymph node metastasis. The results indicate that hypoxic TME play a critical role in activation and pro-tumoral growth by Nrp-1 in cervical cancer [78] (Table 3).

## 4. Biomarkers of TAMs

**Figure 2:**
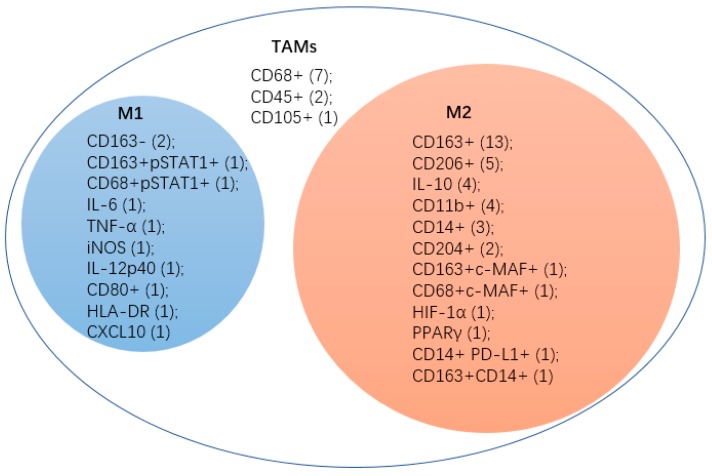
This figure shows the makers which were used in cervical cancer researches. M1, M1-like macrophages; M2, M2-like macrophages; TAMs, tumor-associated macrophages. The numbers in the parentheses represent the number of published papers.

By comparing papers published within the last ten years in the field of macrophage and cervical cancer (Figure 2), we could find that CD68+ was used as a marker to isolate TAMs at an early stage [81,82,83,84,85,86]. Scientists found that CD68+ TAMs in cervical tumor presented a mixed CXCL10 (M1)/CD163+ (M2) pattern [35]. CD45+ and CD105+ are also unspecific for certain subtypes [81,87,88]. Increasing studies have focused on which subtype of macrophages the TAMs belongs to. These studies showed TAMs in CC have a M2-like phenotype because most infiltrating and pro-tumor macrophages were M2-like. M1-like macrophages existed in the CC tumor stroma with a declined expression of M1-like markers such as IL-6, TNF-α and iNOS and tendedto transfer to M2-like phenotype under the influence of tumor cells [27]. Other markers of M1-like were also used in CC research such as CD163- [25,89], CD163+ pSTAT1 [90], IL-12p40 [27], CD80+ [26], HLA-DR [26] and CXCL10 [35]. The most common marker for M2 is CD163+ [25,26,35,73,78,89,91,92,93,94,95,96,97]. CD206+ [26,78,98,99,100] and IL-10 [88,98,100,101]. These are also often used. CD11b+ was considered not very specific for M2-like macrophages. An article pointed out that CD11b+ tumor infiltrating cells may correspond to numerous populations from myeloid-derived suppressor cells to TAMs, or even granulocytes [102]. However, further research has verified that most CD11b+ cells were TAMs with an M2-like phenotype [69] and were adopted by three later studies about CC as an M2-like marker [83,103,104]. CD14+ has been not only used as an M2-like marker in these three articles [84,96,105] but also as the marker of monocytes or macrophages in another article [98]. Many other infrequent markers were also used to recognize M2-like macrophages, such as CD204+ [106], CD163+ c-MAF+ [90], CD68+c-MAF+ [90], HIF-1α [98], PPARγ [98], CD14+PD-L1+ [105] and CD163+CD14+ [28].

## 5. The Switch from M2 to M1-like Macrophages

Since M2-like TAMs are established to exert tumorigenic effect while M1-like TAMs present anti-tumor effect in CC, some researchers have started to focus on the switch from M2- to M1-like macrophages as a possible breakthrough in treatment (Table 4).

Heusinkveld et al. found that upon interaction with CD4^+^ Th1 cells, cervical cancer cell-induced M2-like macrophages could be switched to activated M1-like macrophages that express high levels of costimulatory molecules and acquire the lymphoid homing marker CCR7, thus creating a tumor-rejecting milieu [28]. But the concrete component of Th1 on M2-like macrophages was unclear.

Saito T et al. showed that treatment with recombinant human γ-interferon (rIFN-γ) acted direct dose-dependent inhibition of cervical cancer cell line. Moreover, they also found that human adherent ascites cells (greater than 80% macrophages) e showed strong inhibitory properties towards colony growth of both ovarian carcinoma and melanoma cell lines when treated with rIFN-γ [107]. Although lacking direct evidence, these results suggested that TAMs may produce a diffusible substance under the influence of rIFN-γ to become tumoricidal in CC.

## 6. Development of Therapy by Targeting TAMs

An abundance of molecules has been discovered to act in the formation of TAMs, so the translation of basic research to clinical application is a worthy subject. Here, we will discuss the development of therapy targeting TAMs (Table 5).

### 6.1. Macrophages and Its Prognostic Value in CC

Findings relating to the role of total macrophages in cervical cancer have been controversial. It has been noted that there are higher macrophage counts in invasive carcinomas as opposed to cervical squamous intraepithelial lesions [117]. Moreover, squamous intraepithelial lesions contain more macrophages than normal cervical tissue [118]. In contrast, a study demonstrated that the presence of this prominent infiltration of macrophages did not correlate with the tumor grade or lymph node status but rather showed a strong negative correlation with the tumor stage [119]. However, Davidson et al. proved that macrophage density did not correlate with patient survival in cervical cancer [120]. What is more, some research has proven that CD68 macrophages which represent all activated macrophages were not a prognostic marker [92,106].

The relationship between M2-like macrophages and CC is specific. The number of intra-tumoral M2 TAMs in cervical SCC tissue samples was significantly higher than that of intraepithelial M2-like TAMs in non-tumorous cervical samples. The number of peritumoral M2 TAMs in cervical SCC was higher than in non-tumorous cervical tissues [91]. A high index of CD163+ macrophages was significantly associated with higher FIGO stages and lymph node metastasis [92]. A higher density of tumor infiltrating CD204+ M2 macrophages in uterine cervical adenocarcinoma was significantly associated with shorter disease-free survival [106].

### 6.2. Therapy Targeting the Differentiation of TAMs

Indomethacin (Ind.) is a kind of prostaglandin inhibitor. The combination therapy of OK-432 and Ind. reinforced the M phi-mediated immunopotentiation, resulting in a stronger antitumor effect [108]. In addition, treatment with the COX-inhibitor indomethacin and/or the clinical monoclonal antibody against IL-6R, tocilizumab, prevented M2-like-differentiation [109].

Peritoneal macrophages obtained from patients with CC, when treated with sizofiran (SPG) and rIFN-γ, showed a potent tumoricidal quality, as well as, an increase in the section and the inhibition of the secretion of PGE2. [16].

The synthetic long peptide (SLP) vaccination could induce cytokine-producing T-cells to develop a strong macrophage-skewing capacity, which is necessary for CC tissue shrinkage [110].

### 6.3. Therapy Targeting the Improvement of TAMs Anti-Tumor Activity

SPG-immunotherapy combined with radiotherapy not only induced the cytotoxic activity of macrophages but also augmented NK activity in patients with uterine cervical cancer [111].

Monocytic chemotactic protein-3 (MCP-3) is a CC chemokine originally purified from osteosarcoma cells [121]. In conditions when activated T cells are lacking, heavily infiltrating tumors from hH1/MCP-3-infected HeLa cells with activated macrophages lead to the significant retardation of tumor growth in recipient mice. The result indicates that MCP-3 might activate the phagocytic ability of macrophages [112].

Researchers have found that some HeLa cells produce an uncleavable transmembrane form of TNF (pre-TNF) and that these transformed tumors were compromised in both immunosuppressed and severe combined immunodeficient mice. Macrophages co-cultured with the transformed cells showed increased phagocytosis and cytokine production [113].

Antibodies in serum from a recombinant vaccinia virus expressing the E2 gene of bovine papilloma virus were capable of activating cytotoxicity mediated by infiltrating macrophages for the efficient killing of papilloma tumor cells [114].

Poly methyl methacrylate (PMMA) is a synthetic polymer approved by the Food and Drug Administration for certain human clinical applications such as a bone cement. PMMA 4 particles stimulated the highest level of TNF-α production by macrophages in vitro and yielded the best result of antitumor protection in vivo [115].

A new synthetically prepared fluoroquinolone derivative 6-fluoro-8-nitro-4-oxo-1,4-dihydroquinoline-3-carboxylate (6FN) could induce RAW 264.7 macrophages cell-release of pro- and anti-inflammatory TH1, TH2 and TH17 cytokines with anti-cancer and/or anti-infection activities. A significant inhibition of growth in cancer cells HeLa was detected [116].

## 7. Conclusions

Circulating monocytes are recruited into local lesions and differentiate into two main subtypes of M1-like and M2-like macrophages, which is modulated by various inflammatory cytokines. In cervical cancer, the functional macrophages in tumor progression are the M2-like subtype formed by molecules from CC cells, T-cells, seminal plasma, and an anaerobic microenvironment. Molecules from CC cells play a major role in their differentiation and their alternative activation. HPV oncoproteins, especially E6 and E7, are the initial factor in affecting the expression of these functional molecules. Molecules from the other sources act mainly in the differentiation of monocytes towards the M2-like phenotype. As M1-like macrophages exert an anti-tumor function in CC, the switch of M2 to M1-like macrophages has captured the attention of scientists. How to re-educate the immunosuppressive characteristics of TAMs is still a challenging and valuable problem to be solved.

## Figures and Tables

**Table 1:** The categories and characteristics of macrophages.

Categories	Characteristics
Tissue-resident macrophages	During embryonic organogenesis, they derive from yolk sac and fetal liver precursors [8]. After birth, bone marrow or spleen-derived monocytes can replenish [9]. They show specialized properties and functions to the tissue [9].
Infiltrating macrophages	They derive from circulating monocytes [10]. They present mainly two types of properties M1 and M2 subtypes [10]. M1 is induced by Th1 cytokines and acts in the inflammatory response and antitumor immunity [11]. M2 is induced by Th2 cytokines and acts in the anti-inflammatory and pro-tumorigenic activities [11].

The table above shows the categories of macrophages and their characteristics respectively.

**Table 2:** Tumor-derived molecules acting in tumor-associated macrophage (TAM) formation in cervical cancer (CC).

Categories	Name of Molecules	Potential Mechanism
Molecules promoting the differentiation of monocytes to TAMs	PGE2 and IL-6 [28]	
	Karyopherin β1	Regulating the expression of transcriptional factors NFκB and AP-1 [29]
	CCL2 /MCP-1	Promoting the recruitment of circulating monocytes [30] Enhancing the LPS-induced production of IL-10 [30]
	IL-10	Inhibit the classic activation of macrophages through the JAK1/Tyk2/STAT3 pathway [31]
	PRL	Inducing IL-1β and TNF-α production [32] Inducing the production of anti-inflammatory cytokine IL-10 [33]
Molecules promoting the activation of TAMs	FUCA-1	Affecting the activity of LPS receptor on macrophages [34] Affecting the cytotoxicity of macrophages to tumor cells [34]
	GM-CSF	Promoting TAMs to release pro-tumor factors [35]
	MIF [36]	
	IL-10	Elevating the expression of IL-4Rα [37] Elevating arginase 1 [37]

The table shows tumor-derived molecules and their potential mechanism to form TAMs. PGE2, prostaglandin E2; IL-6, interleukin 6; MCP-1, monocyte chemoattractant protein 1, its alternative name is CCL2; IL-10, interleukin 10; PRL, prolactin; FUCA-1, α-l-Fucosidase; GM-CSF, granulocyte macrophage colony-stimulating factor; MIF, Macrophage migration inhibitory factor; LPS, lipopolysaccharide; TNF-α, tumor necrosis factor α; IL-4R, interleukin 4 receptor.

**Table 3:** Other molecules acting in the formation of TAMs in CC.

Source	Name of Molecules	Potential Mechanism
T cell	IL-17	Increasing the expression of IL-6 [72] Increasing the recruitment of monocytes [72]
	IL-4	Promoting M2a subtype polarization by inducing a transcriptional factor KLF4 [73]
	IL-10	Promoting M2c subtype polarization [44]
	IL-4 and IL-10	Promoting M2a subtype polarization [74]
Seminal plasma		Increasing the expression of COX2, PGE2, IL-6 in CC cells [75,76,77]
Anaerobic microenvironment		Promoting M2 polarization by overexpressing Nrp-1 in CC cells [78]

The table shows the role of the molecules from T cell, seminal plasma and anaerobic microenvironment to the formation of TAMs. IL-17, interleukin 17; IL-6, interleukin 6; KLF4, Kruppel-like factor 4; IL-10, interleukin 10; COX2, cyclooxygenase-2; PGE2, prostaglandin E2; Nrp-1, neuropilin-1.

**Table 4:** Component Involved in the Switch from M2 to M1-like macrophages.

	Component	Potential Mechanism
Targeting the differentiation of TAMs	CD4^+^ Th1 cells	Promoting TAMs to secret costimulatory molecules and the expression of CCR7 [28]
	rIFN-γ	Promoting TAMs to produce a diffusible tumoricidal substance [107]

The table above shows the component involved in the switch from M2 to M1-like macrophages. TAMs, tumor-associated macrophages; rIFN-γ, recombinant human γ-interferon; Th1, 1-type helper T cells.

**Table 5:** Molecules for Therapy by Targeting TAMs.

	Molecules	Potential Mechanism
Targeting the differentiation of TAMs	Ind.	Inhibiting the expression of PG [108]
	tocilizumab	Blocking IL-6R [109]
	rIFN-γ	Simulating the role of IFN-γ to induce M2 to M1-like macrophages [16]
	SLP	Inducing T cells to inflow [110]
Improvement in anti-tumor activity of TAMs	SPG	Inducing the cytotoxic activity of macrophage [111]
	MCP-3	Activating the phagocytic ability of macrophages [112]
	Pre-TNF	Increasing phagocytosis [113]
	bovine papilloma virus antibody	Inducing the cytotoxic activity of macrophage [114]
	PMMA	Stimulating TAMs to produce TNF-α [115]
	6FN	Inducing TAMs to release anti-tumor cytokines [116]

The table above shows the molecules used in therapy by targeting TAMs. Ind., Indomethacin; PG, prostaglandin; rIFN-γ, recombined interferon-γ; IL-6R, interleukin-6 receptor; SLP, synthetic long peptide; SPG, sizofiran; MCP-3, monocyte chemoattractant protein; pre-TNF, an uncleavable transmembrane form of tumor necrosis factor; PMMA, Poly methyl methacrylate; 6FN, 6-fluoro-8-nitro-4-oxo-1,4-dihydroquinoline-3-carboxylate.
